# Perfusion dyssynchrony analysis

**DOI:** 10.1093/ehjci/jev326

**Published:** 2015-12-24

**Authors:** Amedeo Chiribiri, Adriana D.M. Villa, Eva Sammut, Marcel Breeuwer, Eike Nagel

**Affiliations:** ^1^Division of Imaging Sciences and Biomedical Engineering, Department of Cardiovascular Imaging, King's College London, 4th Floor Lambeth Wing, St Thomas’ Hospital, London SE1 7EH, UK; ^2^Philips Healthcare, Imaging Systems—MR Eindhoven, The Netherlands; ^3^Eindhoven University of Technology, Biomedical Engineering, Biomedical Image Analysis, Eindhoven, The Netherlands; ^4^DZHK Centre for Cardiovascular Imaging, University Hospital Frankfurt/Main, Frankfurt am Main, Germany

**Keywords:** perfusion, gadolinium, adenosine, dyssynchrony analysis

## Abstract

**Aims:**

We sought to describe perfusion dyssynchrony analysis specifically to exploit the high temporal resolution of stress perfusion CMR. This novel approach detects differences in the temporal distribution of the wash-in of contrast agent across the left ventricular wall.

**Methods and results:**

Ninety-eight patients with suspected coronary artery disease (CAD) were retrospectively identified. All patients had undergone perfusion CMR at 3T and invasive angiography with fractional flow reserve (FFR) of lesions visually judged >50% stenosis. Stress images were analysed using four different perfusion dyssynchrony indices: the variance and coefficient of variation of the time to maximum signal upslope (V-TTMU and C-TTMU) and the variance and coefficient of variation of the time to peak myocardial signal enhancement (V-TTP and C-TTP). Patients were classified according to the number of vessels with haemodynamically significant CAD indicated by FFR <0.8. All indices of perfusion dyssynchrony were capable of identifying the presence of significant CAD. C-TTP >10% identified CAD with sensitivity 0.889, specificity 0.857 (*P* < 0.0001). All indices correlated with the number of diseased vessels. C-TTP >12% identified multi-vessel disease with sensitivity 0.806, specificity 0.657 (*P* < 0.0001). C-TTP was also the dyssynchrony index with the best inter- and intra-observer reproducibility. Perfusion dyssynchrony indices showed weak correlation with other invasive and non-invasive measurements of the severity of ischaemia, including FFR, visual ischaemic burden, and MPR.

**Conclusion:**

These findings suggest that perfusion dyssynchrony analysis is a robust novel approach to the analysis of first-pass perfusion and has the potential to add complementary information to aid assessment of CAD.

## Introduction

Cardiac magnetic resonance (CMR) has become an established method for myocardial perfusion imaging.^1^ CMR offers superior spatial resolution in comparison to other perfusion imaging modalities. Additionally, an elevated temporal resolution enables the dynamic visualization of the first-pass wash-in of contrast agent in rest and stress conditions.

In normal hearts, the myocardium is perfused relatively homogeneously across all myocardial segments. This results in a homogeneous display of CMR perfusion signals both in the amplitude and in the temporal direction, with the peak myocardial signal intensity occurring nearly simultaneously in all segments a few beats after the peak arterial input signal. In contrast, in ischaemic hearts, abnormal segments display a peak myocardial signal that is both reduced in amplitude and delayed resulting in lower peak signal intensity and temporal dyssynchrony across the ventricle. There have been various approaches to quantification of first-pass perfusion signal intensities; however, to our knowledge, the temporal dyssynchrony of perfusion signals has not yet been exploited directly by any diagnostic algorithm. In this study, we hypothesized that important diagnostic information can be derived by analysing first-pass perfusion signals in the temporal direction.

**Figure 1:**
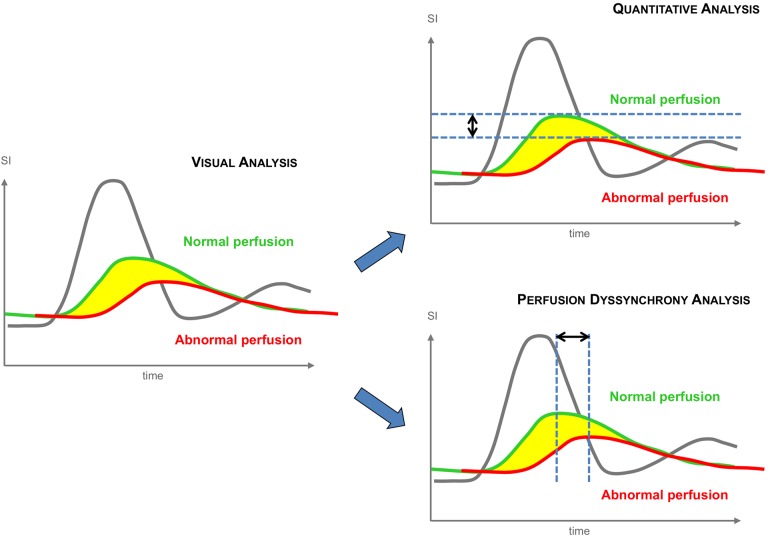
Areas of abnormal myocardial perfusion are characterized by reduced and delayed wash-in of contrast agent. These features are the basis for visual analysis. Quantitative analysis detects and measures absolute differences of perfusion (vertical arrow). To achieve this, myocardial signal intensity curves require temporal realignment before deconvolution with the arterial input function. This is particularly important when high-resolution voxel-wise perfusion quantification is performed. Dyssynchrony analysis instead does not take into account changes in the amplitude of signal intensity but rather isolates and measures the temporal dyssynchrony of the wash-in curves (horizontal arrow). SI, signal intensity.

We sought to describe perfusion dyssynchrony analysis, a novel approach to the analysis of perfusion CMR data. This is specifically designed to isolate and measure the temporal dyssynchrony of myocardial perfusion independently from absolute myocardial blood flow (MBF; *Figure 1*). Specifically, we tested the potential of perfusion dyssynchrony analysis as a tool for the detection of haemodynamically significant coronary artery disease (CAD) assessed by fractional flow reserve (FFR) and to differentiate between patients with single- and multi-vessel CAD.

## Methods

### Study population

Patients referred for stress perfusion CMR were retrospectively included. All patients had undergone invasive coronary angiography and FFR assessment in all vessels with visually >50% severity stenosis within 3 months of the CMR scan. FFR <0.8 was considered haemodynamically significant. Patients were assigned to the Normal, Single-vessel, and Multi-vessel groups according to the results of the invasive assessment. Patients with previous coronary artery bypass grafting, hypertrophic cardiomyopathy, aortic stenosis, or other primary myopathic or valvular disease were excluded. This study was performed in accordance with the principles set by the Declaration of Helsinki and was conducted in accordance with local ethical standards. All participants gave written informed consent.

### CMR acquisition

The CMR scans, including adenosine stress and rest perfusion, functional and scar imaging, were carried out at 3.0T (Philips Achieva-TX, Philips Medical Systems) using standard acquisition protocols.^2^ A *k-t* SENSE gradient echo method was used, and typical sequence parameters were repetition time/echo time 3.0/1.0 ms, flip angle 15°, 90° saturation prepulse, 120 ms prepulse delay, spatial resolution 1.2 × 1.2 × 10 mm^3^. Perfusion data were acquired in three left ventricular (LV) short-axis views covering 16 standard myocardial segments during adenosine-induced hyperaemia over 3 min (140 μg/kg/min) and 15 min later at rest using 0.075 mmol/kg gadobutrol (Gadovist, Schering, Berlin, Germany) at 4 mL/s followed by a 20 mL saline flush. A dual-bolus contrast agent scheme was used as previously described.^3^ Functional data were acquired with steady-state free precession cine sequences prescribed in short axis and long axis of the LV.^4^ Right and LV volumes and function and LV mass were measured according to standard analysis criteria.^5^ Late gadolinium enhancement (LGE) images were acquired 15 min after injection of a top up bolus of contrast agent performed after rest perfusion imaging to a total dose of gadolinium of 0.2 mEq/kg of body weight.^4^

#### Visual CMR analysis

The scans were visually assessed by consensus of at least two expert readers (level of accreditation III according to the guidelines of the Society for Cardiovascular Magnetic Resonance—SCMR) as part of routine clinical assessment.^6,7^ Rest and stress images were reviewed in conjunction with LGE images.^8^ Perfusion defects were defined based on standardized criteria set by the SCMR.^5^ Each cardiac segment was assigned to the appropriate perfusion territory, with segment 15 assigned to the dominant coronary artery (defined by the observer analysing the angiogram).^9^ A visual score was given for image quality of each dataset using a 4-point scale: 1—poor, 2—fair, 3—good, and 4— excellent. The severity of respiratory and dark rim artefacts was also scored on a 4-point and 3-point scale, respectively. For respiratory artefacts: 1—non-diagnostic; 2—severe artefacts but diagnostic; 3—mild artefacts; 4—no artefacts. For dark rim artefacts: 1—circumferential; 2—segmental; 3—absent.

#### Perfusion dyssynchrony analysis

**Figure 2:**
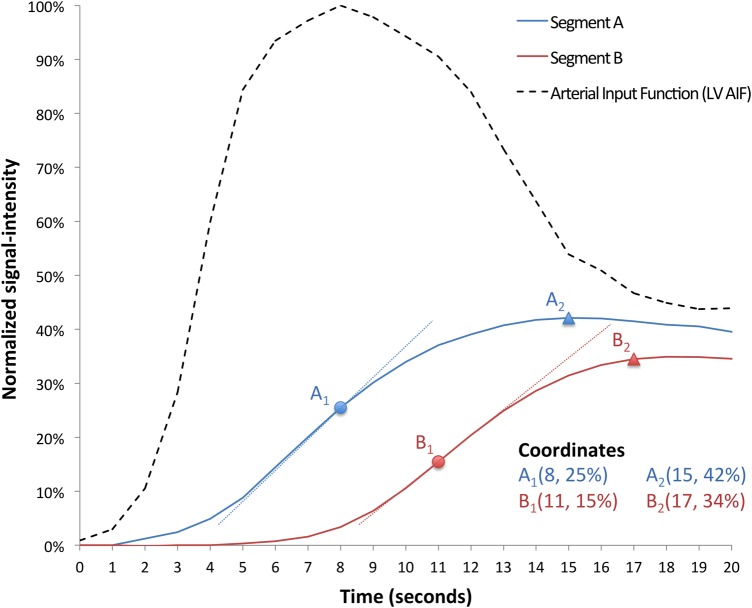
Schematic representation of perfusion dyssynchrony analysis. First-pass myocardial signal intensity curves are shown for two myocardial segments (Segment A and Segment B) and for the AIF measured from the LV cavity. A_1_ and B_1_ indicate the point of maximum signal intensity upslope in each segment. A_2_ and B_2_ indicate the peak of signal intensity in each segment. In this schematic example, the calculated perfusion dyssynchrony indices are coefficient of variation of time to maximum upslope (C-TTMU) 22%; variance of time to maximum upslope (V-TTMU) 2.3 s^2^; coefficient of variation of time to peak signal (C-TTP) 8.8%; variance of time to peak signal (V-TTP) 1 s^2^.

After automated respiratory motion correction and image segmentation,^10^ a grid of 60 angular positions located on chords perpendicular to the myocardial centerline was generated.^11^ Transmural contrast agent wash-in signal intensity curves were then extracted for each angular position and filtered in the spatial and temporal domain using a binomial filter.^12,13^ For each patient, perfusion dyssynchrony analysis was performed on a total of 180 radial segments (60 segments/slice) and on both stress and rest perfusion datasets. The temporal dyssynchrony of LV perfusion was measured as four perfusion dyssynchrony indices; the variance and the coefficient of variation of the time to maximum upslope of the myocardial signal intensity curve (TTMU), and the variance and coefficient of variation of the time to peak myocardial signal intensity (TTP; *Figure 2*). Variances (V-TTMU and V-TTP) are expressed in square seconds (s^2^). Coefficients of variation (C-TTMU and C-TTP) are represented as percentages.

All temporal intervals were calculated starting from the initial upslope of the LV arterial input function (AIF) visually identified by the operator performing the analysis from the basal LV slice. The range of data analysed was set by default to 20 beats from the AIF upslope but could be manually reduced by the operator to avoid respiratory motion occurring towards the end of the acquisition. Perfusion dyssynchrony analysis was repeated twice by the same operator and by a different blinded operator to measure the intra- and inter-observer variabilities.

#### Quantitative perfusion analysis

Quantitative perfusion analysis was performed using Fermi deconvolution according to the methods described by Wilke^14^ and Jerosch-Herold^15^ using in-house software previously validated against positron emission tomography,^16^ FFR,^17^ microspheres,^18^ and hardware perfusion phantom data.^19,20^ Myocardial perfusion reserve (MPR) was defined as the ratio between stress and rest perfusion estimates obtained in each coronary perfusion territory.

#### Catheter laboratory protocol

Invasive coronary angiography was performed with standard methods.^21^ FFR was measured in all vessels that showed visually a >50% diameter stenosis in two orthogonal views during intracoronary adenosine-induced hyperaemia (140 μg/kg/min) with a 0.014-inch coronary pressure sensor–tipped wire (Volcano Therapeutics, San Diego, CA or St Jude Medical, St Paul, MN, USA).^22^

### Statistical analysis

The Medcalc software (Medcalc, Belgium) and Analyse-it software (Analyse-it Software Limited, United Kingdom) were used. Data are presented as mean ± standard deviation. Intra- and inter-observer reproducibilities were determined by Bland–Altman plots and regression analysis. The correlation between perfusion dyssynchrony indices and other quantitative or semi-quantitative CMR parameters was assessed using regression analysis. ANOVA and *t*-tests were used for comparison of results as appropriate. Receiver operating characteristic (ROC) analysis determined the accuracy of each perfusion dyssynchrony index in the diagnosis of CAD and in differentiating between patients with single-vessel and multi-vessel CAD. Optimal diagnostic cut-offs were determined by the best sum of sensitivity and specificity. Mann–Whitney and *χ*^2^ tests were used to test the qualitative measurements for statistical significance. No formal power analysis was carried out.^23^

## Results

A total of 98 subjects were included in the analysis: 35 subjects in the Normal Group, 32 patients in the Single-vessel Group, and 31 patients in the Multi-vessel Group. Baseline data and demographics are shown in *Table 1*. The FFR results are shown in *Table 2*.

**Table 1:** Demographics and risk factors for CAD

	All vessels (*n* = 98)	Normal group (*n* = 35)	Single-vessel group (*n* = 32)	Multi-vessel group (*n* = 31)
Male gender	73 (74%)	21 (60%)	25 (78%)	27 (87%)
Age	60 ± 9	59 ± 10	60 ± 7	62 ± 7
Hypertension	51 (52%)	16 (46%)	19 (59%)	16 (52%)
Dyslipidaemia	65 (66%)	17 (49%)	24 (75%)	24 (77%)
Diabetes	18 (18%)	4 (11%)	9 (28%)	5 (16%)
Current Smoker	15 (15%)	2 (6%)	10 (31%)	3 (10%)
Previous PCI	12 (12%)	0 (0%)	9 (28%)	3 (10%)
Family history of CAD	28 (29%)	4 (11%)	13 (41%)	11 (35%)

CAD, coronary artery disease; PCI, percutaneous coronary intervention.

**Table 2:** FFR results

	All vessels	Normal group	Single-vessel group	Multi-vessel group
Vessels FFR measured	119/294 (40%)	6/105 (6%)	47/96 (49%)	66/93 (71%)
Vessels with FFR >0.8	25/294 (9%)	6/105 (6%)	17/96 (18%)	2/93 (2%)
Vessels with FFR <0.8	94/294 (32%)	0/105 (0%)	30/96 (31%)	64/93 (69%)
FFR negative vessels	0.89 ± 0.06	0.91 ± 0.05	0.88 ± 0.07	0.92 ± 0.09
FFR positive vessels	0.60 ± 0.14	—	0.63 ± 0.14	0.59 ± 0.16

FFR, fractional flow reserve.

### Perfusion CMR

Detailed functional CMR and LGE findings as well as the detailed results of visual assessment and quantitative perfusion analysis are shown in *Table 3*. A total of 294 perfusion territories were included in the analysis. The number of segments visually positive for stress-induced abnormalities was 2.3 ± 2.5 in the Single-vessel and 8.1 ± 3.6 in the Multi-vessel group (*P* < 0.001). There was a significant difference between MPR values in FFR positive and negative perfusion territories (*P* < 0.0001 for all comparisons).

**Table 3:** CMR findings

	All subjects (*n* = 98)	Normal group (*n* = 35)	Single-vessel group (*n* = 32)	Multi-vessel group (*n* = 31)
LV EF (%)	59 ± 6	60 ± 5	59 ± 5	56 ± 7
LV EDV (mL/m^2^)	80 ± 11	74 ± 4	76 ± 6	87 ± 15
LV ESV (mL/m^2^)	35 ± 12	31 ± 5	30 ± 6	38 ± 12
RV EF (%)	55 ± 5	56 ± 5	54 ± 6	56 ± 7
RV EDV (mL/m^2^)	86 ± 10	80 ± 3	90 ± 7	88 ± 16
RV ESV (mL/m^2^)	36 ± 8	34 ± 7	41 ± 8	36 ± 9
LA (cm^2^)	25 ± 5	22 ± 2	24 ± 3	26 ± 4
RA (cm^2^)	22 ± 4	20 ± 3	23 ± 3	24 ± 5
Visual perfusion positive segments (average ± SD per patient)	5.0 ± 4.9	—	2.3 ± 2.5	8.1 ± 3.6
LGE positive segments (average ± SD per patient)	0.5 ± 1.4	—	0.8 ± 1.6	0.9 ± 1.7
MPR (all territories)	2.62 ± 1.1	2.9 ± 1.1	2.8 ± 1.1	2.2 ± 0.8
MPR (territories with FFR <0.8)	1.9 ± 0.6	—	1.9 ± 0.7	1.9 ± 0.6
MPR (territories with FFR >0.8)	2.9 ± 1.1	2.9 ± 1.1	3.0 ± 1.0	2.8 ± 0.8

CAD, coronary artery disease; CMD, coronary microvascular disease; LV, left ventricle; EF ejection fraction; EDV, end-diastolic volume; ESV, end-systolic volume; MPR, myocardial perfusion reserve; RV, right ventricle; LA, left atrium; RA, right atrium; LGE, late gadolinium enhancement; SD, standard deviation; FFR, fractional flow reserve.

#### Perfusion dyssynchrony analysis

Detailed results of perfusion dyssynchrony analysis are shown in *Table 4*. All tested perfusion dyssynchrony indices increased significantly during stress in comparison with rest values, with the exception of the Normal group where perfusion dyssynchrony during stress did not differ from rest values. Moreover, perfusion dyssynchrony values increased proportionally to the extent of haemodynamically significant CAD, with more severe dyssynchrony being induced by adenosine stress in patients with multi-vessel disease. These results were particularly significant when C-TTMU or TTP-derived indices were used. In contrast to perfusion dyssynchrony indices, however, the average TTMU and TTP did not differ between groups and between stress and rest (*Table 4*). Pearson's analysis showed no correlation between average TTMU and TTMU-derived perfusion dyssynchrony indices (*R* = 0.19; *R*^2^ = 0.038 vs. V-TTMU; *R* = 0.31; *R*^2^ = 0.11 vs. C-TTMU). Similarly, no correlation was found between average TTP values and V-TTP or C-TTP (*R* = 0.02; *R*^2^ = 0.001 vs. V-TTP; *R* = 0.33; *R*^2^ = 0.1 vs. C-TTP).

**Table 4:** Comparison between stress and rest values of TTMU, TTP, and perfusion dyssynchrony indices

	Stress	Rest	*P* (stress vs. rest)
V-TTMU			
Normal group	2.7 ± 2.4 s^2^	3.2 ± 3.1 s^2^	0.71
Single-vessel group	3.7 ± 4.0 s^2^	2.8 ± 1.7 s^2^	0.30
Multi-vessel group	4.8 ± 2.8 s^2^	2.5 ± 1.7 s^2^	<0.001
*P* (between groups)	0.03	0.56	
C-TTMU			
Normal group	22 ± 10.1%	24.2 ± 12.7%	0.15
Single-vessel group	27.7 ± 11.2%	22.3 ± 7.3%	0.02
Multi-vessel group	34.1 ± 12.8%	23.4 ± 15.6%	0.003
*P* (between groups)	0.0002	0.89	
V-TTP			
Normal group	1.5 ± 1.1 s^2^	2.2 ± 1.7 s^2^	0.17
Single-vessel group	3.7 ± 3.3 s^2^	2.1 ± 1.1 s^2^	0.01
Multi-vessel group	8.8 ± 6.9 s^2^	2.4 ± 1.3 s^2^	<0.0001
*P* (between groups)	<0.0001	0.07	
C-TTP			
Normal group	8.1 ± 2.9%	8.5 ± 4.3%	0.36
Single-vessel group	14.6 ± 5.2%	10 ± 3.6%	<0.0001
Multi-vessel group	21.7 ± 12.6%	9.6 ± 3.7%	<0.0001
*P* (between groups)	<0.0001	0.40	
Average TTMU			
Normal group	5.6 ± 1.9 s	7.1 ± 3.4 s	0.54
Single-vessel group	6.4 ± 2.7 s	6.5 ± 2.1 s	0.70
Multi-vessel group	6.2 ± 1.9 s	6.9 ± 1.7 s	0.13
*P* (between groups)	0.24	0.67	
Average TTP			
Normal group	14.4 ± 5.1 s	15.2 ± 4.3 s	0.92
Single-vessel group	12.5 ± 3.4 s	12.5 ± 2.4 s	0.95
Multi-vessel group	13.4 ± 3.3 s	14.7 ± 2.3 s	0.06
*P* (between groups)	0.17	0.67	

C-TTMU, coefficient of variation of the time to maximum upslope of the myocardial signal intensity curve; C-TTP, coefficient of variation of the time to peak myocardial signal intensity; V-TTMU, variance of the time to maximum upslope of the myocardial signal intensity curve; V-TTP, variance of the time to peak myocardial signal intensity.

On ROC analysis, perfusion dyssynchrony allowed discrimination between normal subjects and patients with CAD (*Table 5*). TTP-derived indices performed better than TTMU-derived indices. The most accurate parameter for the diagnosis of CAD was C-TTP, with a sensitivity of 0.89, specificity of 0.86, and area under the ROC curve of 0.94. The best C-TTP cut-off for the diagnosis of CAD was >10%. V-TTMU, C-TTMU, and C-TTP were more accurate than visual assessment for the diagnosis of CAD (*P* = 0.0002, *P* = 0.017, and *P* = 0.049, respectively). V-TTMU and C-TTP were more accurate than quantitative analysis for the diagnosis of CAD (*P* = 0.004 and *P* = 0.04, respectively).

**Table 5:** ROC analysis for the prediction of CAD

	Area under ROC curve	95% CI	SE	*Z*	*P*	Best cut-off	Sensitivity	95% CI	Specificity	95% CI
TTMU	0.39	0.27–0.51	0.060	−1.8	0.9639	—	—	—	—	—
V-TTMU	0.63	0.52–0.75	0.059	2.27	0.0117	1.9 s^2^	0.726	0.598–0.831	0.486	0.314–0.660
C-TTMU	0.72	0.61–0.83	0.056	3.96	<0.0001	23%	0.730	0.603–0.834	0.629	0.449–0.785
TTP	0.4	0.28–0.52	0.063	−1.56	0.9410	—	—	—	—	—
V-TTP	0.88	0.81–0.94	0.034	11.15	<0.0001	2.2 s^2^	0.825	0.709–0.909	0.857	0.697–0.952
C-TTP	0.94	0.9–0.98	0.022	20.02	<0.0001	10%	0.889	0.784–0.954	0.857	0.697–0.952
Visual assessment	0.87	0.82–0.93	0.028	13.50	<0.0001	≥1 positive perfusion territory	0.746	0.621–0.847	1	0.900–1.000
Quantitative analysis	0.84	0.76–0.93	0.044	7.77	<0.0001	1.8	0.84	0.727–0.921	0.714	0.537–0.854

C-TTMU, coefficient of variation of the time to maximum upslope of the myocardial signal intensity curve; C-TTP, coefficient of variation of the time to peak myocardial signal intensity; V-TTMU, variance of the time to maximum upslope of the myocardial signal intensity curve; V-TTP, variance of the time to peak myocardial signal intensity.

All perfusion dyssynchrony indices allowed identification of multi-vessel disease (*Table 6*). The most accurate parameter was V-TTP, with a sensitivity of 0.74, specificity of 0.79, and area under the ROC curve of 0.84. The best V-TTP diagnostic cut-off for multi-vessel CAD was >3.3 s^2^. V-TTMU and C-TTMU were more accurate than visual assessment for the diagnosis of multi-vessel disease (*P* = 0.03 for both).

**Table 6:** ROC analysis for prediction of multi-vessel CAD

	Area under ROC curve	95% CI	SE	*Z*	*P*	Best cut-off	Sensitivity	95% CI	Specificity	95% CI
TTMU	0.48	0.36–0.61	0.063	−0.26	0.6044	—	—	—	—	—
V-TTMU	0.68	0.57–0.80	0.058	3.15	0.0008	2.8 s^2^	0.774	0.589–0.904	0.621	0.493–0.738
C-TTMU	0.69	0.58–0.80	0.056	3.43	0.0003	33%	0.516	0.331–0.698	0.776	0.658–0.869
TTP	0.49	0.37–0.61	0.061	−0.23	0.5914	—	—	—	—	—
V-TTP	0.84	0.77–0.92	0.039	8.75	<0.0001	3.3 s^2^	0.742	0.554–0.881	0.791	0.674–0.881
C-TTP	0.81	0.72–0.89	0.043	7.09	<0.0001	12%	0.806	0.625–0.925	0.657	0.531–0.768
Visual assessment	0.84	0.76–0.92	0.043	7.95	<0.0001	≥2 positive perfusion territories	0.710	0.520–0.858	0.970	0.896–0.966
Quantitative analysis	0.79	0.70–0.87	0.045	6.40	<0.0001	1.6	0.903	0.742–0.980	0.657	0.531–0.768

C-TTMU, coefficient of variation of the time to maximum upslope of the myocardial signal intensity curve; C-TTP, coefficient of variation of the time to peak myocardial signal intensity; V-TTMU, variance of the time to maximum upslope of the myocardial signal intensity curve; V-TTP, variance of the time to peak myocardial signal intensity.

Results of average TTMU and TTP were not significant in the prediction of CAD or multi-vessel CAD.

The results of correlation analysis between perfusion dyssynchrony indices, FFR values, visually positive segments, and severity of ischaemia measured as MPR values are shown in *Table 7*. The correlation between perfusion dyssynchrony results and severity of ischaemia was weak, with TTP-derived indices again performing better.

**Table 7:** Results of Pearson's correlation analysis between perfusion dyssynchrony indices and FFR values, number of visually positive segments and MPR

	FFR	Visual analysis^a^	MPR
V-TTMU	*R*^2^ = 0.02 *P* = 0.282	*R*^2^ = 0.04 *P* = 0.047	*R*^2^ = 0.04 *P* = 0.038
C-TTMU	*R*^2^ = 0.04 *P* = 0.093	*R*^2^ = 0.05 *P* = 0.017	*R*^2^ = 0.06 *P* = 0.012
V-TTP	*R*^2^ = 0.12 *P* = 0.004	*R*^2^ = 0.13 *P* < 0.001	*R*^2^ = 0.09 *P* = 0.003
C-TTP	*R*^2^ = 0.10 *P* = 0.008	*R*^2^ = 0.13 *P* < 0.001	*R*^2^ = 0.05 *P* = 0.029

C-TTMU, coefficient of variation of the time to maximum upslope of the myocardial signal intensity curve; C-TTP, coefficient of variation of the time to peak myocardial signal intensity; V-TTMU, variance of the time to maximum upslope of the myocardial signal intensity curve; V-TTP, variance of the time to peak myocardial signal intensity.

^a^Number of positive segments.

**Figure 3:**
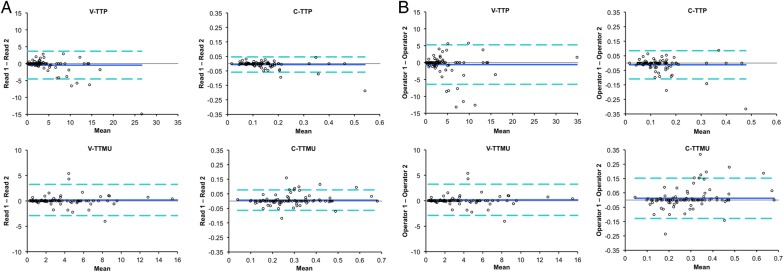
Intra- (*A*) and inter-observer (*B*) variabilities of perfusion dyssynchrony analysis. Bland–Altman graphs for each parameter.

**Figure 4:**
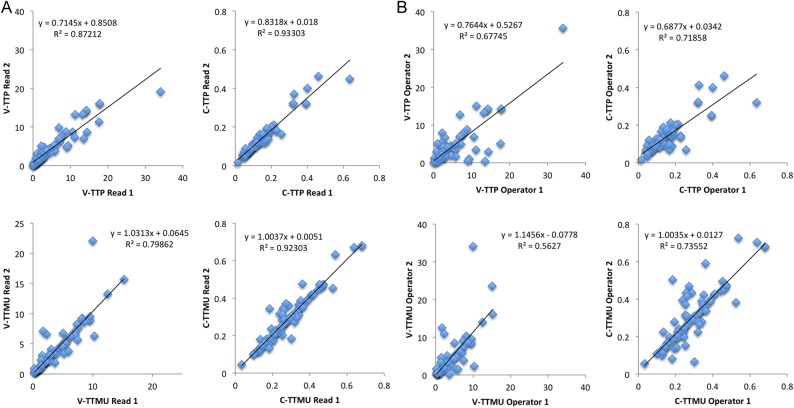
Intra- (*A*) and inter-observer (*B*) variabilities of perfusion dyssynchrony analysis. Pearson's *r* analysis for each parameter.

Bland–Altman graphs and Pearson's *r* analysis for inter- and intra-observer variabilities are shown in *Figures 3* and *4*. C-TTP and C-TTMU were the most reproducible perfusion dyssynchrony indices for intra-operator and inter-operator variabilities.

**Figure 5:**
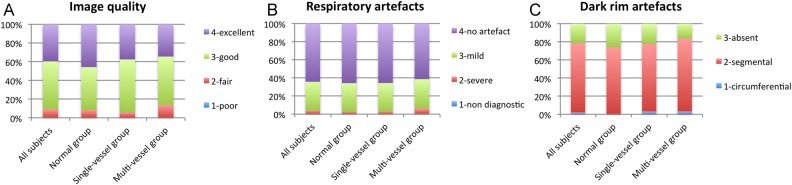
Results of image quality analysis. No significant differences were observed among groups for image quality (*A*), respiratory motion (*B*), and dark rim artefacts (*C*).

Results of overall image qualitative assessment, respiratory artefacts, and dark rim artefacts are presented in *Figure 5*. No significant differences were observed between groups. The average angular extent of dark rim artefact was 35° (range 8–45°).

## Discussion

Perfusion dyssynchrony analysis introduces a novel pathophysiological concept of temporal heterogeneity for the analysis of perfusion CMR data. In this study, we tested the potential of perfusion dyssynchrony analysis to be used as a tool for the detection of CAD and to identify patients with multi-vessel disease.

The main findings of this study are as follows: (i) CAD is associated with temporal dyssynchrony of first-pass perfusion signals, which is proportional to the number of vessels with haemodynamically relevant stenosis as assessed by FFR; (ii) perfusion dyssynchrony is induced by stress; (iii) indices of perfusion dyssynchrony can reliably detect the presence of CAD, and (iv) multi-vessel CAD.

Visual assessment of stress and rest perfusion scans is based on the identification of areas of reduced and delayed wash-in of contrast agent. Quantitative analysis instead measures the absolute reduction myocardial signal intensity without generally accounting for temporal delay, which can potentially be a source of errors,^24^ particularly when voxel-wise techniques are used. We have previously described and validated automated algorithms for correction of the temporal delay for voxel-wise quantification.^25^ However, the observed association between heterogeneous temporal delay and myocardial ischaemia leads us to hypothesize that specific indices of temporal dyssynchrony could be developed and used to detect areas of abnormal perfusion.^25^ To our knowledge, the assessment of myocardial perfusion in term of its temporal component has not yet been explored.

**Figure 6:**
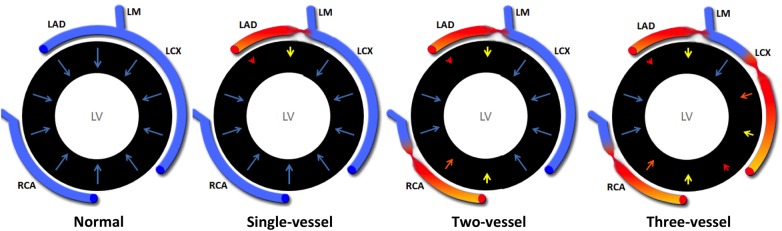
Proposed pathophysiology of myocardial perfusion dyssynchrony. In normal hearts (left), myocardial perfusion shows a temporally homogeneous perfusion. The presence of flow-limiting CAD influences the propagation of the contrast agent through the coronary circulation. Temporal dyssynchrony is caused by the interaction between flow-limiting stenoses and down-stream coronary capacitance. This effect is proportional to the number of flow-limiting coronary lesions. LAD, left anterior descending coronary artery; LCX, left circumflex coronary artery; LM, left main coronary artery; RCA, right coronary artery.

Diastolic blood flow in unobstructed epicardial coronary arteries is very fast, with the dead volume in the epicardial coronaries being replenished several times every heartbeat with fresh blood inflowing from the aorta.^26^ Normal myocardium has preserved vasodilatory reserve and shows a temporally homogeneous perfusion (the myocardium is perfused with uniform amount of blood and wash-in happens at approximately the same time in all segments).^25^ The presence of flow-limiting CAD can deeply influence the propagation of the contrast agent through the coronary circulation, with reduced amplitude and a temporal spread of the signal intensity curves in different coronary territories. Our results demonstrate for the first time that the degree of temporal dyssynchrony correlates with the extent of haemodynamically significant CAD and is maximal in patients with multi-vessel disease. We hypothesized that perfusion dyssynchrony is generated as a result of the interaction between several factors, including the site and severity of the epicardial lesions and the relationship between coronary resistance and down-stream coronary capacitance (*Figure 6*).

Four indices of perfusion dyssynchrony were evaluated in this study, including the variance of the time to maximum myocardial signal upslope (V-TTMU), the coefficient of variation of the time to maximum myocardial signal upslope (C-TTMU), the variance of the time to peak myocardial signal (V-TTP), and the coefficient of variation of the time to peak myocardial signal (C-TTP).

Our results show a progressive increase of perfusion dyssynchrony proportionally to the number of diseased vessels. Importantly, while perfusion dyssynchrony values showed this correlation, the average values of TTMU and TTP did not correlate with the severity of the disease. Moreover, perfusion dyssynchrony indices showed weak correlation with other invasive and non-invasive measurements of the severity of ischaemia, including FFR values (considered as a continuous rather than dichotomous variable), visual ischaemic burden, and MPR values.

All dyssynchrony indices were capable of detecting single- and multi-vessel CAD. However, TTP-derived indices (V-TTP and C-TTP) performed better than TTMU-derived indices. This might reflect the relatively higher signal-to-noise ratio of TTP measures. V-TTP and C-TTP were also the most reproducible indices on both inter- and intra-observer variabilities.

We have previously described and validated transmural perfusion gradient (TPG) analysis against FFR, an another tool to detect stress-induced perfusion abnormalities on high-resolution CMR scans.^13,27^ TPG analysis was designed to take advantage of the high-spatial resolution of CMR, exploiting the differences in first-pass perfusion observed between the inner (sub-endocardial) layers of the LV wall and the outer (sub-epicardial) layers. TPG identifies areas with inducible perfusion abnormalities based on the transmural redistribution of signal during first pass. In principle, TPG analysis is very similar to visual assessment, taking into consideration a combination of amplitude and temporal delay of signal intensity curves at segmental level. Conversely, perfusion dyssynchrony analysis differs from TPG analysis as it does not take into account the amplitude of the signal intensity curves and was rather designed to isolate and measure the temporal dyssynchrony of the wash-in curves to produce one value for the entire left ventricle.

Perfusion dyssynchrony analysis has several potential advantages over other post-processing techniques. It is based on regional differences in the TTMU and TTP rather than on absolute signal intensity values, making it very robust to signal inhomogeneities as well as different data acquisition schemes. In addition, unlike quantitative analysis, perfusion dyssynchrony analysis is not influenced by the complex relationship between contrast agent concentration and signal intensity. This is reflected by low inter- and intra-observer variabilities.

We could find in the literature only very limited attempts to discuss the variability of stress and rest perfusion values in normal subjects by using deconvolution-based quantification algorithms. These results explored the variability of absolute perfusion values rather than the temporal dyssynchrony of the signals.^28^

### Limitations

This study included highly selected populations of patients with suspected CAD. Thus, the data on diagnostic accuracy reflect the accuracy to discriminate between these specific groups rather than a general population. Perfusion dyssynchrony analysis will need to be tested on different scanners, field strength, and with different acquisition protocols. Moreover, patients were included in the study retrospectively, and this constitutes an additional limitation. The presence of coronary microvascular disease or of collateral coronary vessels could in theory affect perfusion dyssynchrony measurements.

## Conclusions

In conclusion, perfusion dyssynchrony analysis is a novel method to measure temporal differences of myocardial perfusion and appears to be highly accurate in identifying patients with haemodynamically significant CAD. In particular, TTP-derived indices showed high accuracy and excellent reproducibility and might represent an additional useful tool in the non-invasive assessment of myocardial ischaemia.

## 

### Funding

Funding to pay the Open Access publication charges for this article was provided by the Engineering and Physical Sciences Research Council (EPSCR).
